# Hepatic resection after transarterial chemoembolization increases overall survival in large/multifocal hepatocellular carcinoma: a retrospective cohort study

**DOI:** 10.18632/oncotarget.13427

**Published:** 2016-11-17

**Authors:** Junwei Chen, Lisha Lai, Qu Lin, Wensou Huang, Mingyue Cai, Kangshun Zhu, Mingsheng Huang

**Affiliations:** ^1^ Department of Radiology, The Third Affiliated Hospital of Sun Yat-Sen University, Guangzhou, 510630 China; ^2^ Department of Interventional Radiology, Ling-nan Hospital, Guangzhou, 510530 China; ^3^ Department of Radiology, Guangzhou First People's Hospital, Guangzhou Medical University, Guangzhou, 510180 China; ^4^ Department of Medical Oncology, The Third Affiliated Hospital of Sun Yat-Sen University, Guangzhou, 510180 China; ^5^ Interventional Radiology Institute, Sun Yat-Sen University, Guangzhou, 510630 China

**Keywords:** hepatocellular carcinoma, transarterial chemoembolization, hepatic resection, prognostic factors

## Abstract

To investigate the prognosis of transarterial chemoembolization (TACE) followed by hepatic resection (HR) in large/multifocal hepatocellular carcinoma (HCC), the medical records of consecutive HCC patients who underwent TACE between January 2006 and December 2010 were retrospectively analyzed. Patients who received TACE alone comprised the T group (61 patients), while those who received HR after TACE comprised the T+R group (49 patients). All the resections were successfully performed, and only one class V complication occurred. While liver function was altered from baseline within 1 week after HR, it recovered within 1 month. Overall survival (OS) of the T+R and T groups were compared, and sub-group analyses were performed based on baseline α-fetoprotein (AFP) levels, the reduction of AFP, and tumor response before HR. Overall survival (OS) in the T+R group was longer than in the T group (47.00 ± 2.87 vs. 20.00 ± 1.85 months, *P* < 0.001). OS in the T+R group with AFP reduction was less than 50%, and OS among those with a poor tumor response before HR did not differ from the T group (*P* > 0.05). These patients may not benefit from the combined treatment. Our findings suggest HR after TACE is safe and effective for large/multifocal HCC, and prolongs OS when compared to TACE alone.

## INTRODUCTION

Hepatocellular carcinoma (HCC) is the 5th most common cancer in the world and the 3rd leading cause of tumor-related deaths [1, 2]. Hepatic resection (HR) is a curative treatment for HCC [3], but the incidence of postoperative recurrence is high [6]. Additionally, approximately 70% of cases are not suitable for curative treatment options because they are diagnosed at non-early stages according to the Barcelona Clinic Liver Cancer (BCLC) staging system [4, 5]. At non-early stages, transarterial chemoembolization (TACE) is the recommended treatment option for HCC [7]. The best candidates for TACE are asymptomatic patients with well-preserved liver function and a solitary or limited multifocal HCC without vascular invasion or extrahepatic spread [8, 9]. However, TACE alone results in incomplete tumor necrosis [10], and the 5-year survival rate is only 6%–19% [11, 12].

Intermediate HCC comprises a highly heterogeneous population that differs according to tumor load, age, and liver function. Bolondi *et al.* [13] proposed a four-stage sub-classification of BCLC-B, and radical therapy was suggested to replace TACE in BCLC-B1 (Table 1). Several studies [14, 15, 16, 17] also suggested that HR was a safe and effective method for intermediate-stage HCC (including BCLC-B1 or BCLC-B2, Table 1). HR combined with TACE was considered the effective method for some intermediate-stage HCC patients, and might improve the effect of the TACE treatment. However, there has been a lack of evidence in whether to perform HR after TACE on non-early HCC patients.

**Table 1:** Four stages sub-classification of BCLC-B

	B1	B2	B3	B4
Child-Pugh	5-6-7	5-6	7	8-9*
Within Ut-7	In	Out	Out	Any
ECOG	0	0	0	0-1
PVT	No	No	No	No
1st Option	TACE	TACE or TARE		Best supportive care
Alternative	Liver Transplantation TACE+ablation	Sorafenib	Research Trail TACE, Sorafenib	Liver Transplantation**

TARE= transarterial radioembolization. Ut-7 = this criterion combines the number of nodules and the size of the largest tumor, with the sum being no more than 7. Examples include two tumors up to 5 cm in size (2 + 5 = 7), three tumors up to 4 cm in size (3 + 4 = 7), etc. A single large tumor up to 6 cm in size (1 + 6 = 7) would also meet the criterion.

* , with severe/refractory ascites and/or jaundice;

** only if Up-to-7 IN and PSO.

We conducted this retrospective study to clarify the role of HR after TACE in patients with large/multifocal HCC lesions. Therapy-related mortality and liver function change were recorded, and long-term survival of those patients treated with TACE+HR was analyzed. In addition, how to determine suitable patients for HR after TACE was further analyzed.

## RESULTS

### Baseline status of patients in our study

**Figure 1:**
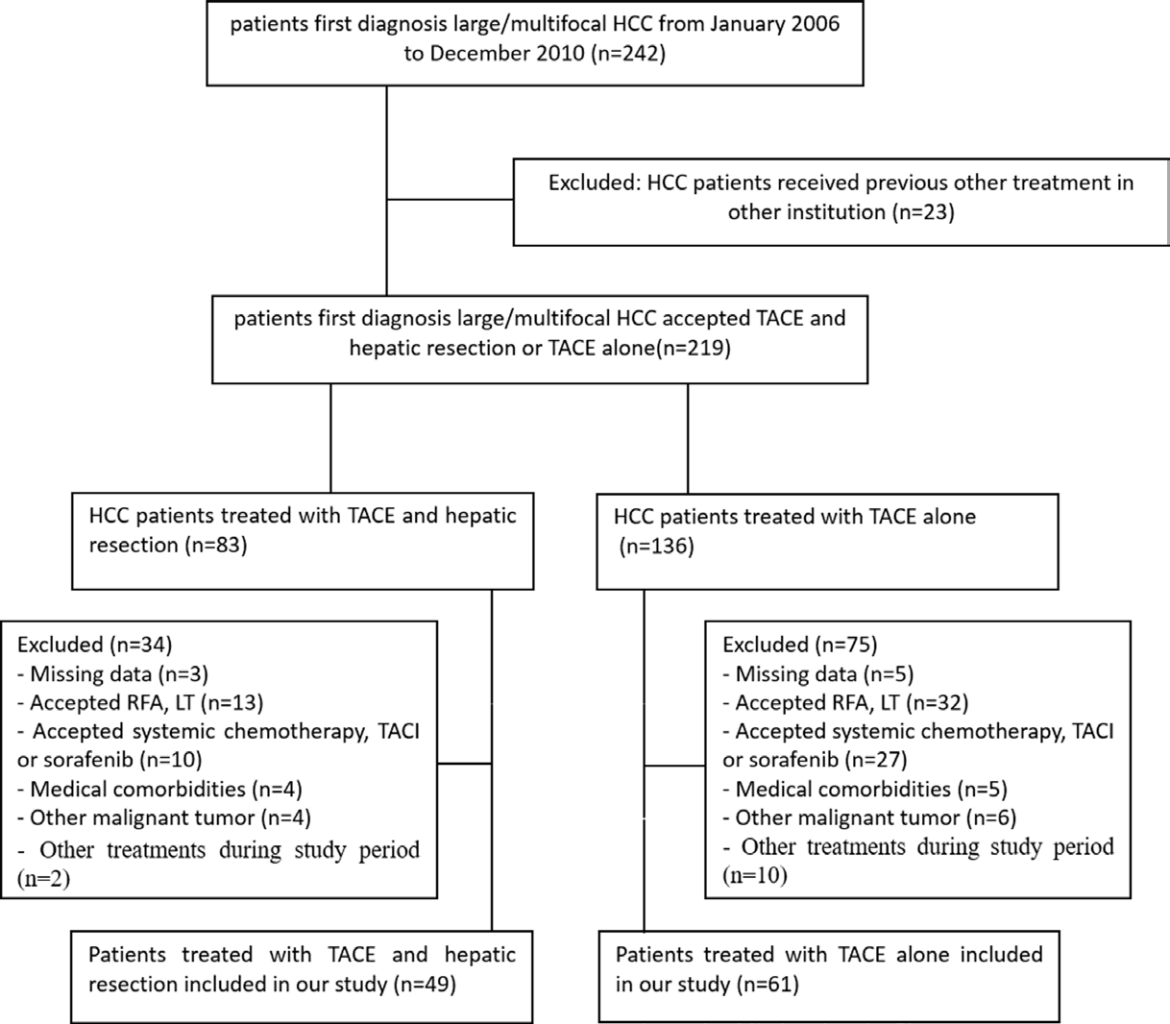
Flow diagram shows exlusion criteria HCC= Hepatocellular carcinoma; TACE= Transarterial chemoembolization; RFA=Radiofrequency ablation; LT=liver transplantation; TACI=transarterial chemoinfusion.

From January 2006 to December 2010, 110 patients with large/multifocal HCC at our institution were included in this study. A total of 132 patients were excluded from the study because they met the exclusion criteria (Figure 1). TACE was recommended as the initial treatment for all 110 patients. A total of 49 patients were also treated with HR (T+R group), leaving 61 patients who received TACE only (T group). The mean follow-up time was 48.2 months (range: 1–101 months) in the T+R group, and 20.9 months (range: 3–67 months) in the T group. The mean number of TACE procedures before HR was 1.74 ± 1.10 (range: 1–4), and the mean time interval between TACE and HR was 3.0 ± 3.2 months in the T+R group.

A comparison of the clinical and demographic characteristics of the patients in these two groups revealed no significant differences (Table 2).

**Table 2:** Baseline character of T+R group and T group

	T+R group	T group	*P*-Value
Age (mean ± SD)	50.31 ± 9.15	51.74 ± 12.06	0.067^#^
Sex			0.764^$^
Male	45(91.8)	58 (95.1)	
Female	4(8.2)	3(4.9)	
Etiology			0.765^%^
HBV	44(89.8)	57(93.4)	
HCV	3(6.1)	2(3.3)	
Other	2(4.1)	2(3.3)	
Child-Pugh			0.884^$^
A	47 (95.9)	57 (93.4)	
B	2 (4.1)	4 (6.6)	
BCLC-B subclassification			0.018^^^
BCLC-B1	17(34.7)	35(57.4)	
BCLC-B2	32(65.3)	26(42.6)	
AFP(ng/ml)			0.784^^^
≥ 200ng/ml	33 (67.3)	45 (73.8)	
< 200ng/ml	16 (32.7)	16 (26.2)	
Tumor diameter (cm)	7.22 ± 3.18	6.80 ± 3.35	0.509^#^
Number(1/2/3/≥ 4)			0.998^^^
1	26 (53.1)	32 (52.5)	
2	15 (30.6)	19 (31.1)	
≥3	8(16.3)	10(16.4)	
Tumor Capsule			0.901^^^
Yes	43(87.8)	54(88.5)	
No	6 (12.2)	7 (11.5)	

Note. - Unless indicated, data are numbers of patients, and numbers in parentheses are percentages

# Independent-samples *t* test was used.

$ Continuity correction was used.

% Fisher exact test was used.

^ Pearson X^2^ test was used.

### Safety of resection after TACE

All of the hepatic resections (100%) in the T+R group were successfully performed. The Clavien-Dindo classification [20] of surgical complications was used to assess post-resection complication. Class I complications occurred in 29 patients, including vomiting (*n* = 13), abdominal pain (*n* = 12), pleural effusion (*n* = 7) and fever (*n* = 11). Class II complications occurred in 4 patients, including bile leakage (*n* = 2) and wound infection (*n* = 2). Only 1 class V complication (gastrointestinal hemorrhage, *n* = 1) occurred after hepatic resection. At 1 week after resection, liver function was altered from baseline, but it recovered in 1 month (Table 3).

**Table 3:** Liver function test at 1 weeks and 1 months after hepatic resection compared to baseline liver function test

	Baseline Value	1 week after resection	*P*-Value	1 month after resection	*P*-Value
**AST**	42.94 ± 17.72	175.62 ± 145.16	0.002	45.25 ± 34.43	0.798
**ALT**	38.06 ± 23.78	179.06 ± 177.97	0.005	46.50 ± 34.03	0.410
**TBILI**	15.06 ± 4.8	26.09 ± 11.59	0.000	15.01 ± 8.12	0.980
**ALB**	42.39 ± 4.42	34.39 ± 6.64	0.000	39.62 ± 4.57	0.036
**PT (sec)**	13.29 ± 0.89	15.51 ± 1.67	0.000	13.61 ± 0.77	0.063

Abbreviations: AST=aspartate aminotransferase; ALT=alanine aminatransferase; TBILI=Total bilirubin; ALB=albumin; PT=prothrombin time.

### Overall survival and the 1-yr, 2-yr, and 3-yr survival rates

**Figure 2:**
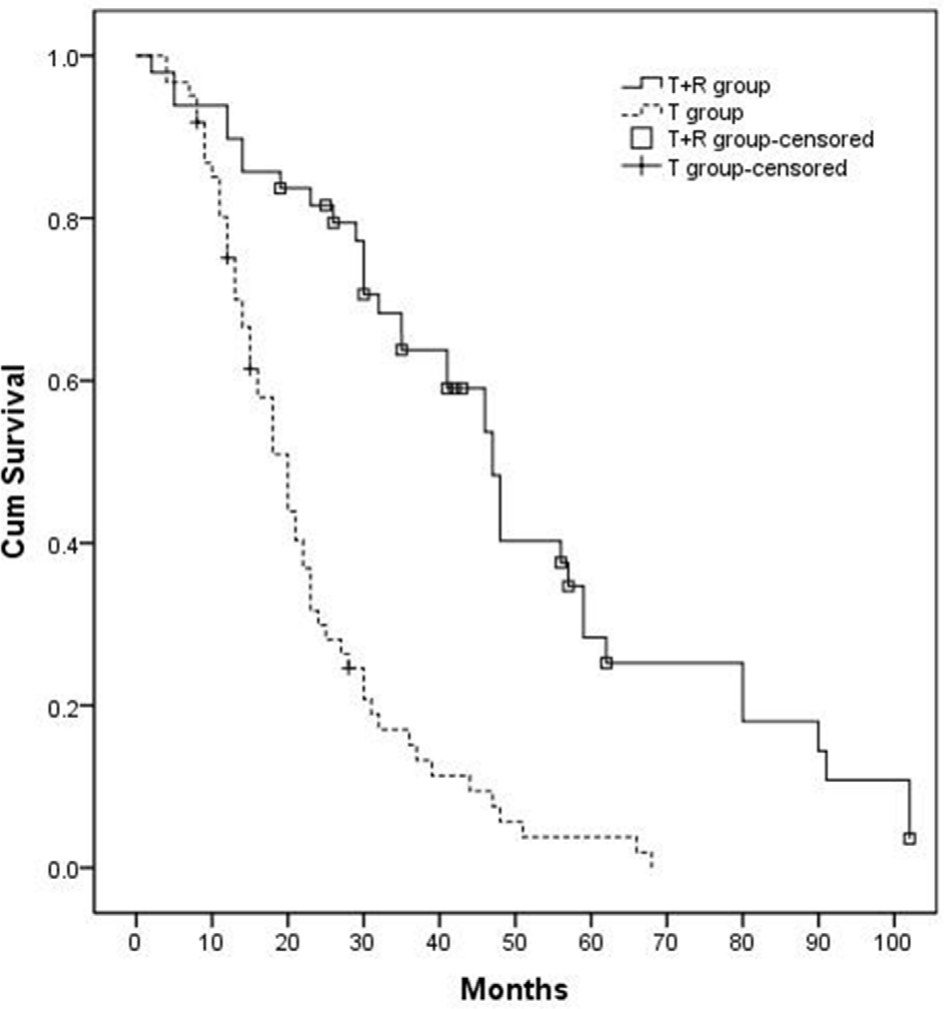
Kaplan-Meier curves of OS in patients with large/multifocal HCC who underwent TACE-resection (T+R group) or TACE (T group) The OS of T+R group (*n* = 49) was 47.0 months, and the OS of T group (*n* = 61) was 20.0 months (*P* < 0.001).

The median OS time in the T+R group and T group were 47.0 (95% CI 41.4–52.6) months and 20.0 (95% CI 16.4–23.6) months, respectively (*P* < 0.001, Figure 2). The 1-, 2-, and 3- year survival rates in the T+R group were 89.8%, 79.4%, 59.1%, respectively; the 1-, 2-, and 3- year survival rates in the T group were 75.1%, 61.5%, and 15.1%, respectively.

### Univariate and multivariate analysis

Univariate analyses revealed that T+R treatment method, single tumor lesion, good tumor response, and lower baseline AFP were associated with better OS. These 4 factors were selected as candidates for multivariate analysis. The multivariate Cox proportional hazard model revealed that T + R treatment method (HR = 0.24, 95% CI = 0.151–0.395. *P* < 0.001), good tumor response (HR = 0.27, 95% CI = 0.160–0.469. *P* = 0.043) and lower baseline AFP (HR = 0.59, 95% CI = 0.366–0.965. *P* < 0.001) were identified as independent prognostic factors for OS (Table 4).

**Table 4:** Univariate analysis and multivariate analysis of OS

Factors	No. of Patients	Median OS	*P*-Value
Turmor No.			0.012*
1	30	48.0 (38.3–57.7)	
≧ 1	19	41.0 (30.7–51.3)	
mRECIST before resection			0.021*
Good Response	37	48.0 (45.9–50.1)	
Poor Response	12	35.0 (14.3–55.7)	
Baseline AFP			0.059*
< 200 ng/ml	16	47.0 (12.3–22.8)	
≧ 200 ng/ml	33	46.0 (39.7–52.3)	
Reduction of AFP^&^			0.011^^^
> 50%	22	48.0 (43.6–52.5)	
< 50%	11	19.0 (5.3–32.7)	

Note-Data in parentheses are 95% CIs.

* Log-rank test was used.

& Only 33 patients with baseline AFP value ≥ 200 ng/ml were included in the univariate analyses.

^ Breslow test was used.

### Sub-group analysis according to tumor response before hepatic resection

**Figure 3:**
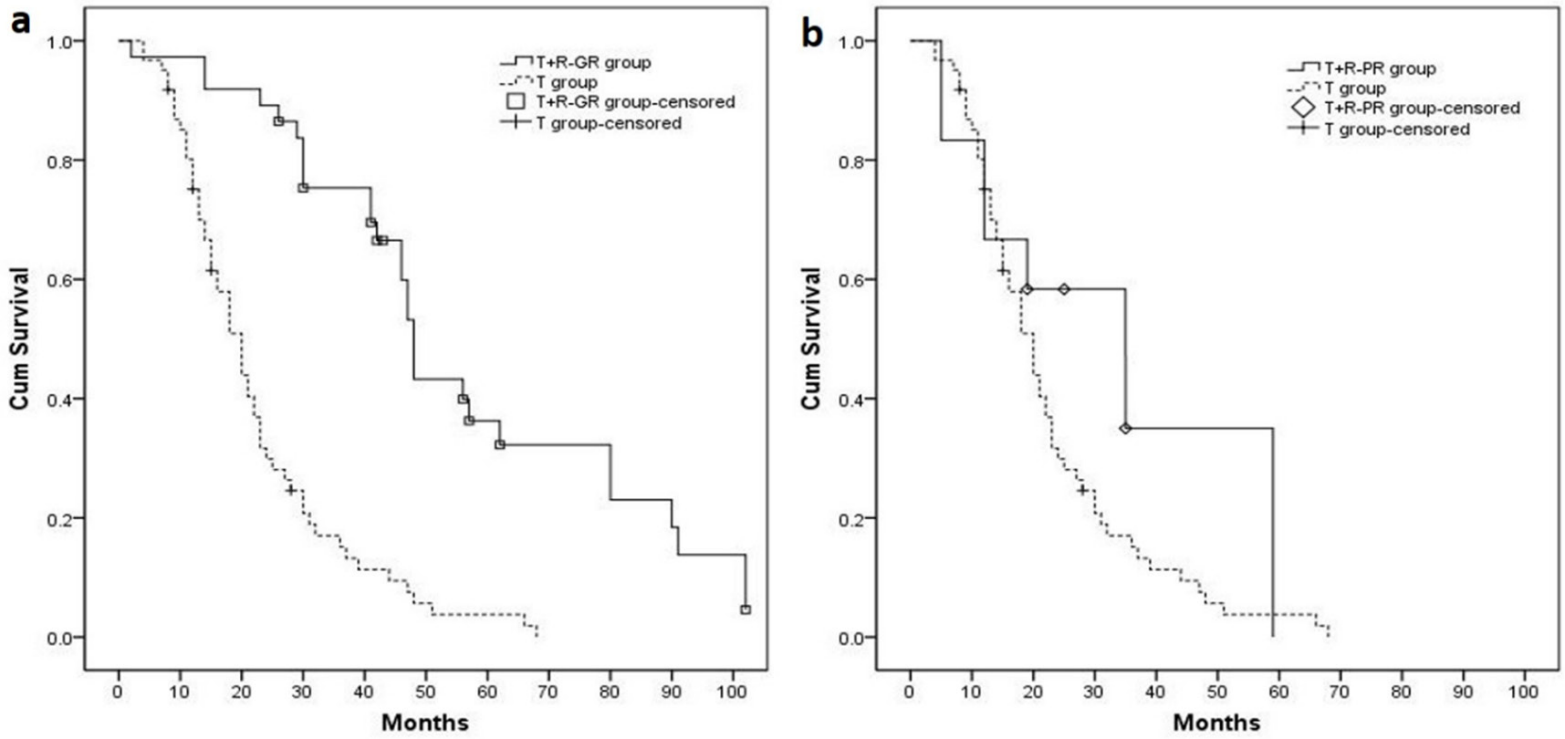
(**A**) Patients with good response before hepatic resection (T+R–GR group, *n* = 37, median OS = 48.0 months) had longer median OS compared with T group (*P* < 0.001). (**B**) Patients with poor response before hepatic resection (T+R–PR group, *n* = 12, median OS = 35.0 months) had no significant difference with T group (*P* = 0.135).

Modified RECIST (mRECIST) criteria was used to sub-categorize patients prior to HR. Patients with complete response (CR) or partial response (PR) were defined as the T+R_-GR_ group; patients with stable disease (SD) or progressive disease (PD) were defined as the T + R_-PR_ group. Thirty-seven (37) patients in the T+R group exhibited good response, including 14 patients with CR and 23 patients with PR. Only 12 patients did not exhibit a good response, including 6 patients with SD and 6 patients with PD. The median OS time of the T+R_-GR_ and T+R_-PR_ groups were 48.0 (95% CI 45.9–50.1) months and 35.0 (95% CI 14.3–55.7) months. OS of these 2 sub-groups were compared to the T group (Figure 3A: T+R_-GR_ group *vs.* T group: *P* < 0.001; Figure 3B: T+R_-PR_ group vs. T group: *P* = 0.135.).

### Sub-group analysis according to baseline AFP level

**Figure 4:**
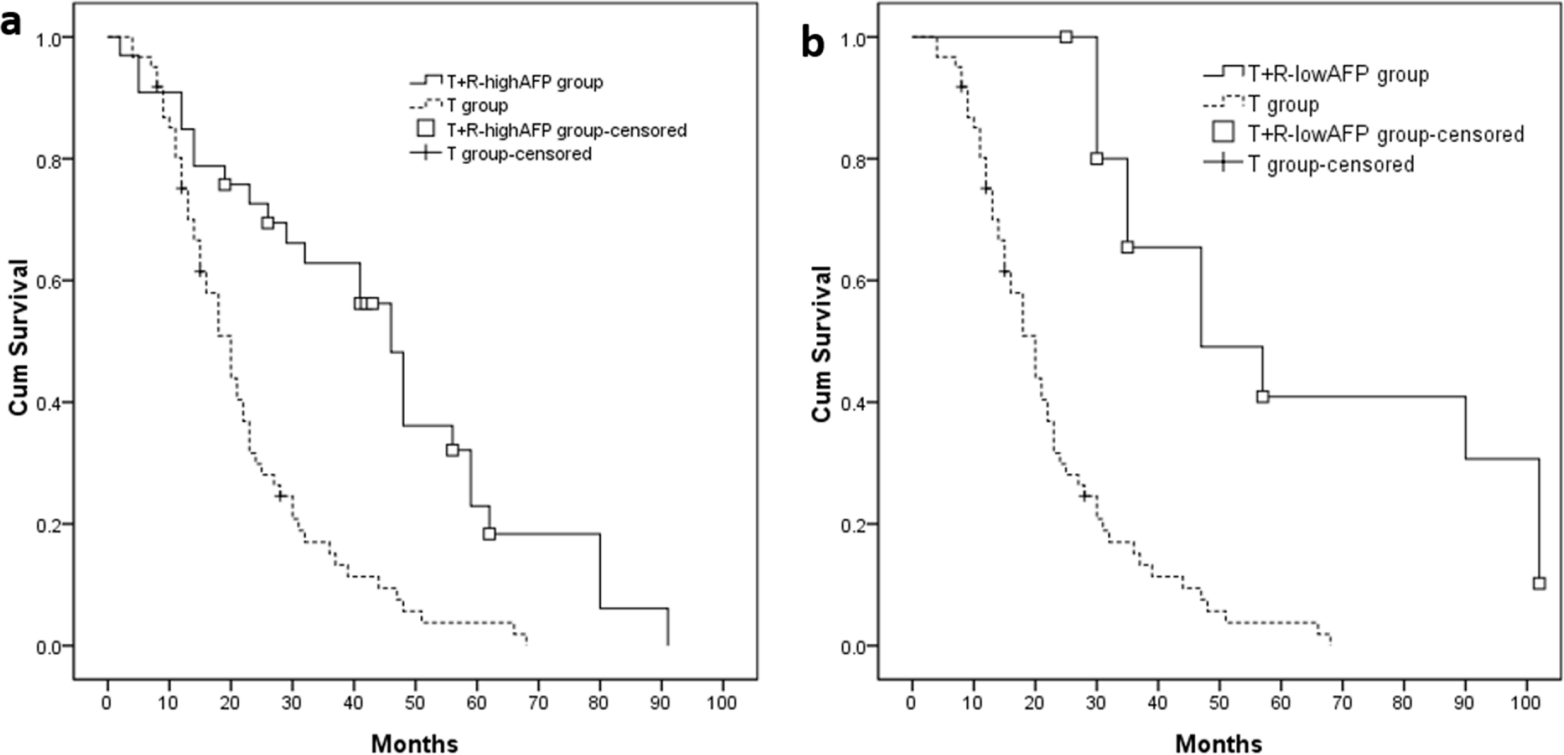
(**A, B**) Patients with high baseline AFP value (T+R-highAFP group, *n* = 33, median OS = 46.0 months, *P* < 0.001) or low baseline AFP value (T+R-lowAFP group, *n* = 16, median OS = 47.0 months, *P* < 0.001) had longer median OS compared with T group.

Thirty-three (33) patients with a high baseline AFP level (≥ 200 ng/ml) in the T+R group were defined as the T+R_-highAFP_ group, and 16 patients with low baseline AFP level (< 200 ng/ml) were defined as the T+R_-lowAFP_ group. The median OS of T+R_-highAFP_ group and T+R_-lowAFP_ group were 46.0 (95% CI 39.7–52.3) months and 47.0 (95% CI 22.8–71.2) months. OS of these 2 sub-groups were compared to the T group (Figure 4A: T+R_-highAFP_ group *vs.* T group: *P* < 0.001; Figure 4B: T+R_-lowAFP_ group *vs.* T group: *P* < 0.001).

### Sub-group analysis according to AFP reduction before hepatic resection

**Figure 5:**
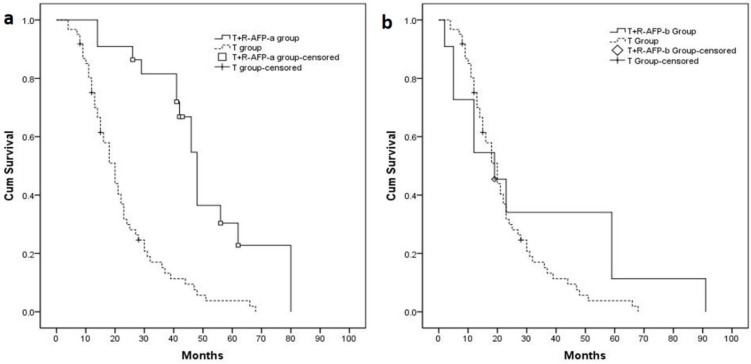
(**A**) Patients in the T+R_**-AFP-a**_ group (*n* = 22, median OS = 48.0 months) had longer median OS compared with T group (*P* < 0.001). (**B**) Patients in the T+R_-AFP-b_ group (*n* = 12, median OS = 19.0 months) had no significant difference with T group (*P* = 0.247).

Twenty-two (22) patients that experienced an AFP level decrease > 50% before resection were defined as the T+R_-AFP-a_ group; 11 patients whose AFP reduction was < 50% were defined as the T+R_-AFP-b_ group. The median OS of the T+R_-AFP-a_ and T+R_-AFP-b_ groups were 48.0 (95% CI 43.6–52.5) months and 19.0 (95% CI 5.3–32.7) months. The median OS of these 2 sub-groups were compared to the T group (Figure 5A: T+R_-AFP-a_ group vs. T group: *P* < 0.001; Figure 5B: T+R_-AFP-b_ group vs. T group: *P* = 0.247).

## DISCUSSION

Large/multifocal HCC is typically treated with TACE [8]. The 1-, 2- and 3-year survival rates of these patients have historically only been 75%, 47%, and 26%, respectively [21], and we found similar results in this retrospective study. HR may be one treatment option when patients did not show a complete response following TACE [24], and sequential resections may prolong survival [22, 23]. However, few studies have examined the safety and long-term efficacy of patients who underwent HR following TACE.

Due to advances in HR techniques and peri/post-operative care, HR for HCC can be performed without mortality, even in cirrhotic patients [25]. In our study, the success rate of resection after TACE was 100%, and only 1 class V complication occurred after resection. AST, ALT, TBILI, ALB and PT-sec levels were altered within 1 week after resection (*P* < 0.05), but liver function recovered in 1 month (*P* > 0.05, except the ALB level). We concluded that HR could be safely performed after TACE in patients with large/multifocal HCC.

The main limitations of TACE are incomplete necrosis due to the dual blood supply around the capsule, multiple collateral circulation, or recanalization of the embolized artery [26]. Furthermore, patients with large HCC rarely achieve complete remission by undergoing TACE alone. To overcome these limitations and eliminate residual cancer cells left behind by TACE, a combined treatment regimen of HR following TACE was performed at our institution. The median OS of patients who underwent TACE and HR treatment was longer than that of patients who underwent TACE alone (47.0 months vs. 20.0 months, *P* < 0.05). Moreover, our data showed that the 1-, 2- and 3- year survival rates in the T+R group were higher than those of patients who underwent HR alone (89.8%, 79.4%, 59.1% vs. 83%, 62%, 49%) [27]. Therefore, HR following TACE is suggested to be more safe and effective for large/multifocal HCC patients.

It was difficult to select suitable patients for the combined HR + TACE treatment. We assumed that tumor response after TACE, baseline AFP value, and reduction of AFP level after TACE might be independent prognosis factors that impact the survival in patients who underwent HR after TACE.

Detection of arterial lesions by enhanced CT/MR was used to evaluate viable tumors according to mRECIST [19, 28]. mRECIST is correlated with long-term survival [29, 30], and it was used to determine the amount of tumor necrosis induced by TACE. Furthermore, Lei's study [31] suggested that mRECIST may represent selection criterion for intermedian-HCC for resection. Adachi E *et al.* [32] reported that preoperative TACE resulted in better disease-free survival rates when complete tumor necrosis was induced. However, other studies [33, 34] have found that the extent of tumor necrosis was actually not a favorable prognostic factor. In this study, patients with good tumor response in the T+R group experienced longer OS than those in the T group (*P*<0.001). In contrast, the OS of patients with poor tumor response in the T+R group was not significantly different with that of the T group (*P* = 0.135). We presumed that the tumor response before HR might be a valuable prognostic factor for large/multifocal HCC patients.

AFP values correlated with tumor activity, and it has been shown that AFP is an important determinant of the degree of malignancy of HCC in cytological studies [35, 36, and 37]. A close relationship exists between the level of serum AFP and HCC recurrence/metastasis [38, 39]. AFP values have been considered a significant independent predictor of large/multifocal patients [40]. We presumed that a reduction in the AFP level after TACE might indicate the preoperative TACE had reduced tumor activity, which in turn might lead to better results in the following resection procedure. In our study, patients with AFP reduction > 50% before HR showed longer OS than TACE alone (*P* < 0.001). In contrast, patients with AFP reduction < 50% did not show a difference in OS from the T group (*P* = 0.247).

There were several limitations of our study. First, our study was retrospective, and therapeutic options (TACE-HR vs. TACE alone) in patients with large/multifocal HCC were individually determined on the basis of the attending physician's preference. Second, the numbers of patients were limited, especially in the T+R_-PR_ (12 patients) and T+R_-AFP-b_ groups (11 patients). The low patient numbers in these groups might have led to the observed differences in OS. We suggest that an adequately powered, prospective, randomized trial of TACE combined with resection is needed for further study.

In conclusion, HR following TACE can be safely performed and is more effective than TACE alone. Large/multifocal HCC patients with poor tumor response, higher baseline AFP level, and AFP decrease less than 50% before HR might not be suitable for HR after TACE.

## MATERIALS AND METHODS

### Study design and patient selection

This study protocol was approved by the ethics committee of our hospital and written informed consent was obtained from the patients. We reviewed the electronic medical records of 242 consecutive patients diagnosed with intermediate HCC from January 2006 to December 2010 (all follow-ups were completed by December 2014) at the third affiliated Hospital of Sun Yat-sen University. HCC was diagnosed according to noninvasive criteria in accordance with the European Association for the Study of the Liver/American Association for the study of Liver Disease guidelines.

The inclusion criteria for the study population were as follows: (a) patients between 18 and 75 years of age, (b) an ECOG performance status of 0, (c) Child-Pugh class A or B liver disease, and (d) single large HCC (> 5 cm) or multifocal HCC without vascular invasion or extra-hepatic spread [12, 17]. Patients were excluded from this study if they had any of the following: (a) previously undergone local-regional therapies (radiofrequency ablation, percutaneous ethanol injection, or iodine 125 seed implantation), hepatic resection, and liver transplantation as an initial treatment, or TACE at other institutions; (b) underwent sorafenib therapy, systemic chemotherapy, or transarterial chemoinfusion (TACI) during our study; (c) underwent other treatment methods (radiofrequency ablation, percutaneous ethanol injection, or iodine 125 seed implantation, et al.) in addition to TACE+HR or TACE during this study; (d) had serious medical comorbidities; or (e) currently had or had a history of malignant tumors in addition to HCC.

### Transarterial chemoembolization and hepatic resection procedure

The clinical treatment strategy of HCC patients was determined by a multidisciplinary liver tumor conference at our hospital. Large/multifocal HCC patients who received TACE as their initial treatment were included in this study. Patients were suggested to undergo HR after 1 – 4 TACE procedures, and the indications for surgery in our department were as follows: (a) a Child-Pugh A/B classification, total bilirubin < 51.30 μmol/L, serum total protein > 60 g/L or serum albumin > 30 g/L, and a prothrombin time ≤ 17 s; (b) no obvious presence of hepatic decompensation; (c) anatomic resection was our preferred surgical method for hepatic resection for multiple nodules in one segment or in neighboring segments; (d) an appropriate residual liver volume evaluated by CT; and (e) no contraindications for major surgery indicated by a multidisciplinary evaluation. Patients in accordance with these indications were suggested to receive HR after TACE. If patients and their relatives agreed to perform HR, resection would be performed at our institute by 4 surgeons who had 10–15 years of experience in the procedure. Patients who refused HR underwent TACE alone.

TACE was performed with a 5-French catheter (Cook, Bloomington, Indiana, USA) or microcatheter (Renegade, Boston Scientific, Natick, MA, USA, or Progreat, Terumo, Tokyo, Japan) as selectively as possible through the lobar, segmental, or subsegmental arteries, depending on the tumor distribution and hepatic functional reserve. Initially, an emulsion of lipiodol (Lipiodol Ultrafluido, Guerbet, France) and doxorubicin hydrochloride was administered into the feeder vessels. The volume of lipiodol ranged from 2 to 20 ml, and the amount of doxorubicin ranged from 20 to 60 mg. 300–500 μm gelatin sponge particles (Cook, Bloomington, Indiana, USA) were mixed with contrast material, and then administered into the feeder vessels until stasis of the arterial flow was achieved. A solution of lobaplatin at a concentration of 0.5 mg/ml was infused into the tumor feeder vessels at a rate of 5 ml/min. The total amount of lobaplatin used ranged from 20 to 50 mg depending on the patient's body weight.

Hepatic resection was performed under general anesthesia via an L-shaped laparotomy or bilateral subcostal incision with a midline extension. Intraoperative ultrasound (US) was routinely performed to evaluate the tumor burden, liver remnant, and the resection margin. An intermittent Pringle's maneuver [18] for 20 min and a 5-minute clamp-free interval were used to reduce blood loss during resection. Two Jackson–Pratt drains (size 10) were placed after surgery.

### Follow-up and repeated TACE

The clinical, laboratory, and radiologic records were reviewed. Additionally, laboratory liver function tests, including serum total bilirubin, albumin and prothrombin time, at 1 week and 1 month after HR were used to evaluate the safety of HR after TACE.

The tumor response was evaluated with contrast-enhanced CT/MR imaging according to the modified Response Evaluation Criteria in Solid Tumors (mRECIST) [19]. Baseline tumor measurements were performed in both groups. In the T+R group, CT/MR exam was performed at 1 month after resection and every 3 months thereafter. During the follow-up period, patients in both groups with recurrent tumor, according to contrast-enhanced CT/MR images, underwent repeated TACE if the Child-Pugh status remained at class A or B and there was no evidence of hepatic decompensation (e.g., uncontrolled ascites or hepatic encephalopathy). Contrast-enhanced CT/MR was performed at 1 month after the initial TACE procedure in the T group, and again every 1 – 2 months. Further TACE therapy was based on mRECIST on contrast-enhanced CT/MR imaging, and decided by consensus.

Furthermore, we compared and analyzed the overall survival (OS) between the T+R group and the T group for the total study population. OS was defined as the time from the date of the first TACE procedure until death or the last follow-up.

### Statistical analysis

All statistical analyses were performed using SPSS version 16.0 (SPSS Inc, Chicago, IL, USA). Pearson chi-square tests, continuity correction, independent-samples *t*-tests, and Fisher's exact tests were used to determine significant differences between the groups. A Wilcoxon signed rank test was used to determine the difference in liver function test values before and after resection. The OS was calculated for both groups using Kaplan-Meier methods. Univariate analyses were performed by the log-rank test, and variables with a *P* value of less than 0.05 at univariate analysis were entered into a multivariate analysis. Multivariate analysis by Cox proportional hazards model was performed to identify independent prognostic factors. A *P*-value of less than 0.05 was considered significant.
